# Impact of malnutrition on mortality and neurological recovery of older patients with spinal cord injury

**DOI:** 10.1038/s41598-024-56527-y

**Published:** 2024-03-11

**Authors:** Koji Tamai, Hidetomi Terai, Hiroaki Nakamura, Noriaki Yokogawa, Takeshi Sasagawa, Hiroaki Nakashima, Naoki Segi, Sadayuki Ito, Toru Funayama, Fumihiko Eto, Akihiro Yamaji, Kota Watanabe, Junichi Yamane, Kazuki Takeda, Takeo Furuya, Atsushi Yunde, Hideaki Nakajima, Tomohiro Yamada, Tomohiko Hasegawa, Yoshinori Terashima, Ryosuke Hirota, Hidenori Suzuki, Yasuaki Imajo, Shota Ikegami, Masashi Uehara, Hitoshi Tonomura, Munehiro Sakata, Ko Hashimoto, Yoshito Onoda, Kenichi Kawaguchi, Yohei Haruta, Nobuyuki Suzuki, Kenji Kato, Hiroshi Uei, Hirokatsu Sawada, Kazuo Nakanishi, Kosuke Misaki, Akiyoshi Kuroda, Gen Inoue, Kenichiro Kakutani, Yuji Kakiuchi, Katsuhito Kiyasu, Hiroyuki Tominaga, Hiroto Tokumoto, Yoichi Iizuka, Eiji Takasawa, Koji Akeda, Norihiko Takegami, Haruki Funao, Yasushi Oshima, Takashi Kaito, Daisuke Sakai, Toshitaka Yoshii, Tetsuro Ohba, Bungo Otsuki, Shoji Seki, Masashi Miyazaki, Masayuki Ishihara, Seiji Okada, Shiro Imagama, Satoshi Kato

**Affiliations:** 1 Department of Orthopaedic Surgery, Osaka Metropolitan University Graduate School of Medicine, 1-5-7 Asahimachi, Abenoku, Osaka, Osaka 545-8585 Japan; 2 Department of Orthopaedic Surgery, Graduate School of Medical Sciences, Kanazawa University, 13-1 Takara-machi, Kanazawa, Ishikawa 920-8641 Japan; 3 Department of Orthopaedic Surgery, Toyama Prefectural Central Hospital, 2-2-78 Nishinagae, Toyama, Toyama 930-8550 Japan; 4 Department of Orthopedic Surgery, Nagoya University Graduate School of Medicine, 65 Tsurumai-cho, Showa-ku, Nagoya, 466-8550 Japan; 5 Department of Orthopaedic Surgery, Faculty of Medicine, University of Tsukuba, 1-1-1 Tennodai, Tsukuba, Ibaraki 305-8575 Japan; 6 Department of Orthopaedic Surgery, Graduate School of Comprehensive Human Sciences, University of Tsukuba, 1-1-1 Tennodai, Tsukuba, Ibaraki 305-8575 Japan; 7 Department of Orthopaedic Surgery, Ibaraki Seinan Medical Center Hospital, 2190, Sakaimachi, Ibaraki, Sashima 306-0433 Japan; 8 Department of Orthopaedic Surgery, Keio University School of Medicine, 35 Shinanomachi, Shinjuku-ku, Tokyo, 160-8582 Japan; 9 Department of Orthopaedic Surgery, National Hospital Organization Murayama Medical Center, 2-37-1 Gakuen, Musashimurayama, Tokyo, 208-0011 Japan; 10 Department of Orthopaedic Surgery, Japanese Red Cross Shizuoka Hospital, 8-2 Otemachi, Aoi-ku, Shizuoka, 420-0853 Japan; 11 Department of Orthopaedic Surgery, Graduate School of Medicine, Chiba University, 1-8-1 Inohana, Chuo-ku, Chiba, Chiba 260-8670 Japan; 12 Department of Orthopaedics and Rehabilitation Medicine, Faculty of Medical Sciences, University of Fukui, 23-3 Matsuoka Shimoaizuki, Eiheiji-cho, Yoshida-gun, Fukui, 910-1193 Japan; 13 Department of Orthopaedic Surgery, Hamamatsu University School of Medicine, 1-20-1, Handayama, Higashi-ku, Hamamatsu, Shizuoka 431-3192 Japan; 14 Department of Orthopaedic Surgery, Nagoya Kyoritsu Hospital, 1-172 Hokke, Nakagawa-ku, Nagoya-shi, Aichi 454-0933 Japan; 15 Department of Orthopaedic Surgery, Sapporo Medical University, South 1-West 16-291, Chuo-ku, Sapporo, 060-8543 Japan; 16 Department of Orthopaedic Surgery, Matsuda Orthopedic Memorial Hospital, North 18-East 4-1 Kita-ku, Sapporo, 001-0018 Japan; 17 Department of Orthopaedic Surgery, Yamaguchi University Graduate School of Medicine, 1-1-1 Minami-Kogushi, Ube, Yamaguchi 755-8505 Japan; 18 Department of Orthopaedic Surgery, Shinshu University School of Medicine, 3-1-1 Asahi, Matsumoto, Nagano 390-8621 Japan; 19 Department of Orthopaedics, Graduate School of Medical Science, Kyoto Prefectural University of Medicine, Kawaramachi-Hirokoji, Kamigyo-ku, Kyoto, 602-8566 Japan; 20 Department of Orthopaedics, Saiseikai Shiga Hospital, 2-4-1 Ohashi, Ritto, Shiga 520-3046 Japan; 21 Department of Orthopaedic Surgery, Tohoku University Graduate School of Medicine, 1-1 Seiryo-machi, Aoba-ku, Sendai, Miyagi 980-8574 Japan; 22 Department of Orthopaedic Surgery, Graduate School of Medical Sciences, Kyushu University, 3-1-1 Maidashi Higashi-ku, Fukuoka, 812-8582 Japan; 23 Department of Orthopaedic Surgery, Nagoya City University Graduate School of Medical Sciences, 1 Kawasumi, Mizuho-cho, Mizuho-ku, Nagoya, 467-8601 Japan; 24 Department of Orthopaedic Surgery, Nihon University Hospital, 1-6 Kanda-Surugadai, Chiyoda-ku, Tokyo, 101-8393 Japan; 25 Department of Orthopaedic Surgery, Nihon University School of Medicine, 30-1 Oyaguchi Kami-cho, Itabashi-ku, Tokyo, 173-8610 Japan; 26 Department of Orthopedics, Traumatology and Spine Surgery, Kawasaki Medical School, 577, Matsushima, Kurashiki, Okayama 701-0192 Japan; 27 Department of Orthopaedic Surgery, Kitasato University School of Medicine, 1-15-1, Kitazato, Minami-ku, Sagamihara, Kanagawa 252-0374 Japan; 28 Department of Orthopaedic Surgery, Kobe University Graduate School of Medicine, 7-5-1 Kusunoki-cho, Chuo-ku, Kobe, 650-0017 Japan; 29 Department of Orthopaedic Surgery, Kochi Medical School, Kochi University, Kohasu, Oko-cho, Nankoku, 783-8505 Japan; 30 Department of Orthopaedic Surgery, Graduate School of Medical and Dental Sciences, Kagoshima University, 8-35-1 Sakuragaoka, Kagoshima, 890-8520 Japan; 31 Department of Orthopaedic Surgery, Gunma University Graduate School of Medicine, 3-39-22 Showa, Maebashi, Gunma 371-8511 Japan; 32 Department of Orthopaedic Surgery, Mie University Graduate School of Medicine, 2-174 Edobashi, Tsu, Mie 514-8507 Japan; 33 Department of Orthopaedic Surgery, School of Medicine, International University of Health and Welfare, 852 Hatakeda, Narita, Chiba 286-0124 Japan; 34 Department of Orthopaedic Surgery, International University of Health and Welfare Narita Hospital, 852 Hatakeda, Narita, Chiba 286-0124 Japan; 35 Department of Orthopaedic Surgery and Spine and Spinal Cord Center, International University of Health and Welfare Mita Hospital, 1-4-3 Mita, Minato-ku, Tokyo, 108-8329 Japan; 36 Department of Orthopaedic Surgery, The University of Tokyo Hospital, 7-3-1 Hongo, Bunkyo-ku, Tokyo, 113-8655 Japan; 37 Department of Orthopaedic Surgery, Osaka University Graduate School of Medicine, 2-2 Yamadaoka, Suita, Osaka 565-0871 Japan; 38 Department of Orthopedics Surgery, Surgical Science, Tokai University School of Medicine, 143 Shimokasuya, Isehara, Kanagawa 259-1193 Japan; 39 Department of Orthopaedic Surgery, Tokyo Medical and Dental University, Yushima 1-5-45, Bunkyo-ku, Tokyo, 113-8519 Japan; 40 Department of Orthopaedic Surgery, University of Yamanashi, 1110 Shimokato, Chuo, Yamanashi 409-3898 Japan; 41 Department of Orthopaedic Surgery, Graduate School of Medicine, Kyoto University, 54 Shogoin-Kawaracho, Sakyo-ku, Kyoto, Kyoto Japan; 42 Department of Orthopaedic Surgery, Faculty of Medicine, University of Toyama, 2630 Sugitani, Toyama, Toyama 930-0194 Japan; 43 Department of Orthopaedic Surgery, Faculty of Medicine, Oita University, 1-1 Idaigaoka, Hasama-machi, Yufu-shi, Oita 879-5593 Japan; 44 Department of Orthopaedic Surgery, Kansai Medical University Hospital, 2-3-1 Shinmachi, Hirakata, Osaka 573-1191 Japan

**Keywords:** Trauma, Outcomes research

## Abstract

This retrospective cohort study established malnutrition’s impact on mortality and neurological recovery of older patients with cervical spinal cord injury (SCI). It included patients aged ≥ 65 years with traumatic cervical SCI treated conservatively or surgically. The Geriatric Nutritional Risk Index was calculated to assess nutritional-related risk. Overall, 789 patients (mean follow-up: 20.1 months) were examined and 47 had major nutritional-related risks on admission. One-year mortality rate, median survival time, neurological recovery, and activities of daily living (ADL) at 1 year post-injury were compared between patients with major nutrition-related risk and matched controls selected using 1:2 propensity score matching to adjust for age, pre-traumatic neurological impairment, and activity. In the Kaplan–Meier analysis, the median survival times were 44.9 and 76.5 months for patients with major nutrition-related risk and matched controls, respectively (p = 0.015). Matched controls had more individuals with a neurological improvement of American Spinal Injury Association Impairment Scale ≥ 1 grade (p = 0.039) and independence in ADL at 1 year post-injury than patients with major nutrition-related risk (p < 0.05). In conclusion, 6% of older patients with cervical SCI had major nutrition-related risks; they showed a significantly higher 1 year mortality rate, shorter survival time, poorer neurological improvement, and lower ADL at 1 year post-injury than matched controls.

## Introduction

The incidence of spinal cord injury (SCI) is currently reported to be as high as 3.6 to 195.4 per million worldwide. Notably, the direct and indirect costs of SCI are higher than those of similar conditions, such as dementia, multiple sclerosis, and cerebral palsy^1,2^. Furthermore, the current global aging is spurring the burden of SCI on the population and social economy^3–5^. The proportion of the world’s population aged > 60 years is expected to nearly double from 12 to 22% between 2015 and 2050^6^. The older population is known to have high rates of osteoporosis, degenerative changes in the spine, and falls due to declines in functional ability^7,8^. In fact, a high increase rate in SCI incidence from 84 to 131 cases/million (average annual percentage change: 2.7%) was observed in the older population in the United States between 1993 and 2012^9^. For such a population, minor traumas, including low-velocity falls, are becoming the major mechanism of underlying traumatic SCI^3–5,10^. Due to its characteristic injury pattern and background, 90% of SCIs reportedly occur in the cervical spine rather than in the thoracic spine among the older population^5^.

Malnutrition among older individuals is a major social concern worldwide. A recent large-scale survey conducted by the Japanese government revealed that 17% of the older population in Japan experienced malnutrition^11^. Therefore, since nutritional status is a critical factor for patients with SCI, understanding the influence of malnutrition on the outcomes after SCI is necessary. Previous studies have elucidated the relationship between malnutrition in the general population with SCI and clinical complications/mortality^12–14^. However, the influences of malnutrition in older populations with SCI on clinical outcomes, including mortality and neurological recovery, remains unclear.

Furthermore, a concern exists that the parameters of nutritional status are not unified throughout these studies and that this causes difficulty in interpreting and comparing results^11–14^. The Geriatric Nutrition Risk Index (GNRI) is a simple index of nutrition-related risk that focuses on the evaluation of older patients^15^. This index has recently been shown to be an objective and reliable tool for assessing nutrition-related risk in various pathological conditions in the older population^16–19^. Therefore, this study aimed to determine nutrition-related risk and its impact on mortality and neurological recovery of older patients with cervical SCI using GNRI.

## Methods

### Study design and ethical considerations

This study retrospectively analyzed multicenter registry data collected by the Japan Association of Spine Surgeons with Ambition (JASA)^20–22^. All study participants provided written informed consent. The Institutional Review Board of the representative facility reviewed and approved this study (Approval No.: 3352-1). All methods were performed in accordance with the principle of the Declaration of Helsinki and Ethical Guidelines for Medical and Health Research Involving Human Subjects in Japan. No funding was received for this study.

### JASA database

The JASA members, who are spine surgeons from 78 institutions in Japan, reviewed the medical records of their institutions and retrospectively registered the cases in the JASA database based on the following inclusion and exclusion criteria^20–22^. The following patients were included those aged ≥ 65 years with traumatic cervical SCI, those treated conservatively or surgically between 2010 and 2020 at one’s institution, and those followed up for at least 3 months post-injury. The exclusion criteria were cervical metastases and missing data.

### Data collection

#### Patient background data

Data regarding age at injury, sex, height, weight, body mass index (BMI), number of patients with diabetes, pre-injury ADL (independent, able to walk with assistance, or wheelchair/bedridden), number of ventilator dependents due to SCI-induced respiratory dysfunction, blood examination results at the first visit, and survival data at 1-year post-injury were collected. Blood tests included those for hemoglobin (g/dL), total protein (g/dL), and Alb (g/dL). ADL data on admission and at 1 year post-injury were extracted from the database.

#### Neurological impairment scale

American Spinal Injury Association Impairment Scale (ASIA) Impairment Scale (AIS) which is International standards of neurologic classification of SCI was used as a parameter of neurological impairment^23^: Grade A: complete impairment; Grade B: sensory incomplete impairment; Grade C: motor incomplete impairment; Grade D: motor incomplete impairment; and Grade E = no neurological impairment. AIS data on admission and at 1 year post-injury were extracted from the database.

#### Therapeutic data

Treatment strategies for patients, including conservative or surgical treatment, were recorded. All treatments were determined on a case-by-case basis by each attending physician.

### Geriatric Nutrition Risk Index

The GNRI was calculated from the patient’s serum Alb, weight, and ideal weight using the following formula: GNRI = [1.489 × Alb (g/L)] + [41.7 × (body weight/ideal body weight)]^15^. The ideal body weight was defined as the value calculated from height and BMI of 22 kg/m^2^^16^ Body weight/ideal body weight was set to 1 if the body weight exceeded the ideal body weight^15^. The GNRI has the following grading system: > 98 = absence of nutrition-related risk; 92 to ≤ 98 = low risk; 82 to < 92 = moderate risk; and < 82 = major risk.

### Statistical analysis

#### Overview analysis

The overall distribution of nutrition-related risk among patients was narratively described. Subsequently, patients were categorized into two groups using the GNRI data. GNRI was calculated from the data on admission, and patients with GRNI < 82 were defined as those with major nutrition-related risks. All other patients were allocated to the control group. Patient demographics between the groups were compared using the Mann–Whitney U or Chi-square test as appropriate. The results of the residual analysis were considered significant at p values < 0.05 when the variable showed |r|> 1.96, in accordance with the Haberman method^24^.

#### Propensity score matching

A matched control group was created using propensity score matching. Therefore, to estimate the propensity score, we fitted a logistic regression model using age, sex, pre-injury ADL, pre-injury AIS grade, and treatment. The nearest-neighbor 1:2 matching procedure was used, restricting the matched propensities to be within 0.01 units of each other.

#### Survival time analysis

The mortality rate within 1 year was compared between patients with major nutrition-related risk and matched controls using the chi-square test. Kaplan–Meyer analysis was used to calculate the survival curve and median survival time with 95% CI post-trauma. Finally, the log-rank test was used to compare the results between patients with major nutrition-related risks and matched controls.

#### Neurological improvement and ADL analysis

The number of patients whose neurological symptoms improved by ≥ 1 AIS grade was compared between patients with major nutrition-related risk and matched controls. In this analysis, we compared the numbers after stratification according to the AIS grade on admission^20^, and the ADL at 1 year post-injury were also compared between the groups.

#### Settings

Continuous variables are presented with a mean ± 1.0 standard deviation. All analyses were performed using IBM SPSS Statistics for Windows, version 26.0 (IBM Corp., Armonk, N.Y., USA). Statistical significance was set at p values < 0.05.

### Ethical approval

The institutional review board of the representative facility reviewed and approved this study. (Kanazawa University, No. 3352-1).

## Results

### Overview

In total, 789 patients were enrolled in this study (mean age: 75.2 ± 6.6 years; 567 males and 225 females; mean follow-up: 20.1 ± 21.7 months). Among all patients, 47 (6.0%), 226 (28.7%), and 516 (65.4%) had a GNRI of < 82 (indicating major nutrition-related risk), 82–98 (indicating moderate to low risk), and > 98 (indicating no risk), respectively (Table 1). When comparing patients with major nutrition-related risk and controls, the proportion of females was significantly higher, BMI was significantly lower, pre-traumatic ADL was significantly lower, and all blood test results were significantly lower in patients with major nutrition-related risk than in controls (Table 2, p = 0.016, < 0.001, 0.023, and < 0.001, respectively).

**Table 1:** Nutrition-related risk of all patients.

Nutrition-related risk	GNRI	Numbers (%)
Major risk	< 82	47 (6.0)
Moderate risk	82 to < 92	107 (13.6)
Low risk	92 to < 98	119 (15.1)
No risk	≥ 98	516 (65.4)

*GNRI* Geriatric Nutritional Risk Index.

**Table 2:** Overall comparisons of demographics between patients with and those without major nutrition-related risk.

	Patients with major nutrition-related risk	Patients without major nutrition-related risk	p value
Numbers	47	742	
Age (years) ± SD	76.5 ± 7.8	75.1 ± 6.6	0.242*
Female/male	15/32	107/535	0.016^#^
BMI (kg/m^2^) ± SD	17.8 ± 2.1	22.5 ± 3.5	< 0.001*
Pre-trauma ADL (%)			0.023^#^
Independent	36 (76.6)	660 (88.9)	< 0.05^‡^
Walk with assistance	10 (21.3)	67 (9.0)	< 0.05^‡^
Wheelchair/bedridden	1 (2.1)	15 (2.0)	
Blood test data			
TP (g/dL) ± SD	5.6 ± 0.5	6.6 ± 0.6	< 0.001*
Alb (g/dL) ± SD	2.6 ± 0.4	3.8 ± 0.6	< 0.001*
Hb (g/dL) ± SD	10.7 ± 2.3	12.9 ± 1.8	< 0.001*
AIS grade			
A	7 (14.9)	85 (11.5)	0.436^#^
B	5 (10.6)	49 (6.7)	
C	17 (36.2)	239 (32.5)	
D	18 (38.3)	363 (49.3)	
Treatment			
Conservative	16 (34.0)	256 (34.5)	1.000^#^
Surgical	31 (66.0)	486 (65.4)	

*SD* standard deviation, *BMI* body mass index, *ADL* activities of daily living, *TP* total protein, *Alb* albumin, *Hb* hemoglobin, *AIS* American spinal injury association impairment scale.

*Mann–Whitney U test, ^#^Chi-square test, ^‡^residual analysis.

### Matching

After 1:2 propensity score matching, 94 patients were selected as matched controls. No significant differences were found in age, sex ratio, number of patients with diabetes, pre-traumatic ADL, AIS grade, or number of ventilator-dependent or treatment procedures between patients with major nutrition-related risk and matched controls (Table 3, p = 0.818, 0.858, 0.421, 0.980, 1.000, and 0.534 respectively).

**Table 3:** Comparisons of demographics after matching procedures.

	Patients with major nutrition-related risk	Matched control	p value
Numbers	47	94	
Age (years) ± SD	76.5 ± 7.8	76.9 ± 7.4	0.818*
Female/male	15/32	32/62	0.851^#^
Diabetes	10 (21.3)	26 (27.7)	0.539^#^
Pre-trauma ADL (%)			
Independent	36 (76.6)	80 (85.1)	0.421^#^
Walk with assistance	10 (21.3)	12 (12.8)	
Wheelchair/bedridden	1 (2.1)	2 (2.1)	
Follow-up (month) ± SD	16.6 ± 12.0	22.1 ± 12.1	0.099
AIS grade			
A	7 (14.9)	12 (12.8)	0.980^#^
B	5 (10.6)	9 (9.6)	
C	17 (36.2)	35 (37.2)	
D	18 (38.3)	38 (40.4)	
Ventilator dependent	5 (10.6)	7 (7.4)	0.534
Treatment			
Conservative	16 (34.0)	32 (34.0)	1.000^#^
Surgical	31 (66.0)	62 (66.0)	
BMI (kg/m^2^) ± SD	17.8 ± 2.1	23.1 ± 4.2	< 0.001*
Blood test data			
TP (g/dL) ± SD	5.6 ± 0.6	6.5 ± 0.7	< 0.001*
Alb (g/dL) ± SD	2.6 ± 0.4	3.7 ± 0.6	< 0.001*
Hb (g/dL) ± SD	10.7 ± 2.3	12.7 ± 1.7	< 0.001*

*SD* standard deviation, *BMI* body mass index, *ADL* activities of daily living, *TP* total protein, *Alb* albumin, *Hb* hemoglobin, *AIS* American spinal injury association impairment scale.

*Mann–Whitney U test; ^#^Chi-square test.

### Survival analysis

**Figure 1:**
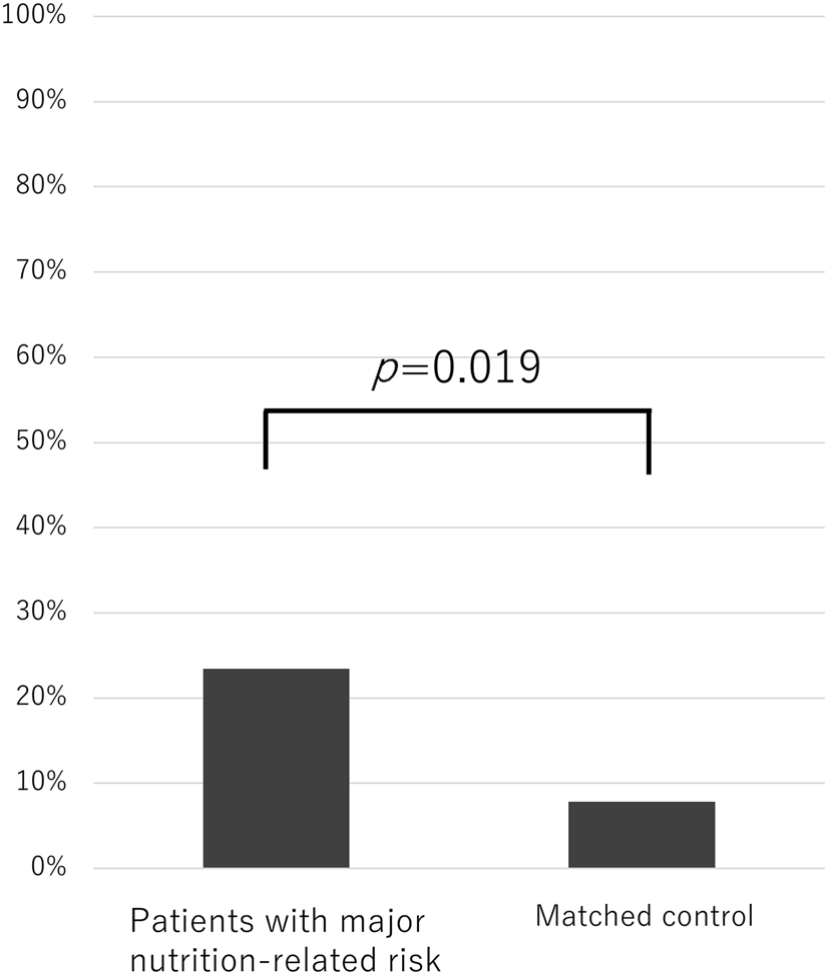
Mortality rate within 1 year post-injury.

**Figure 2:**
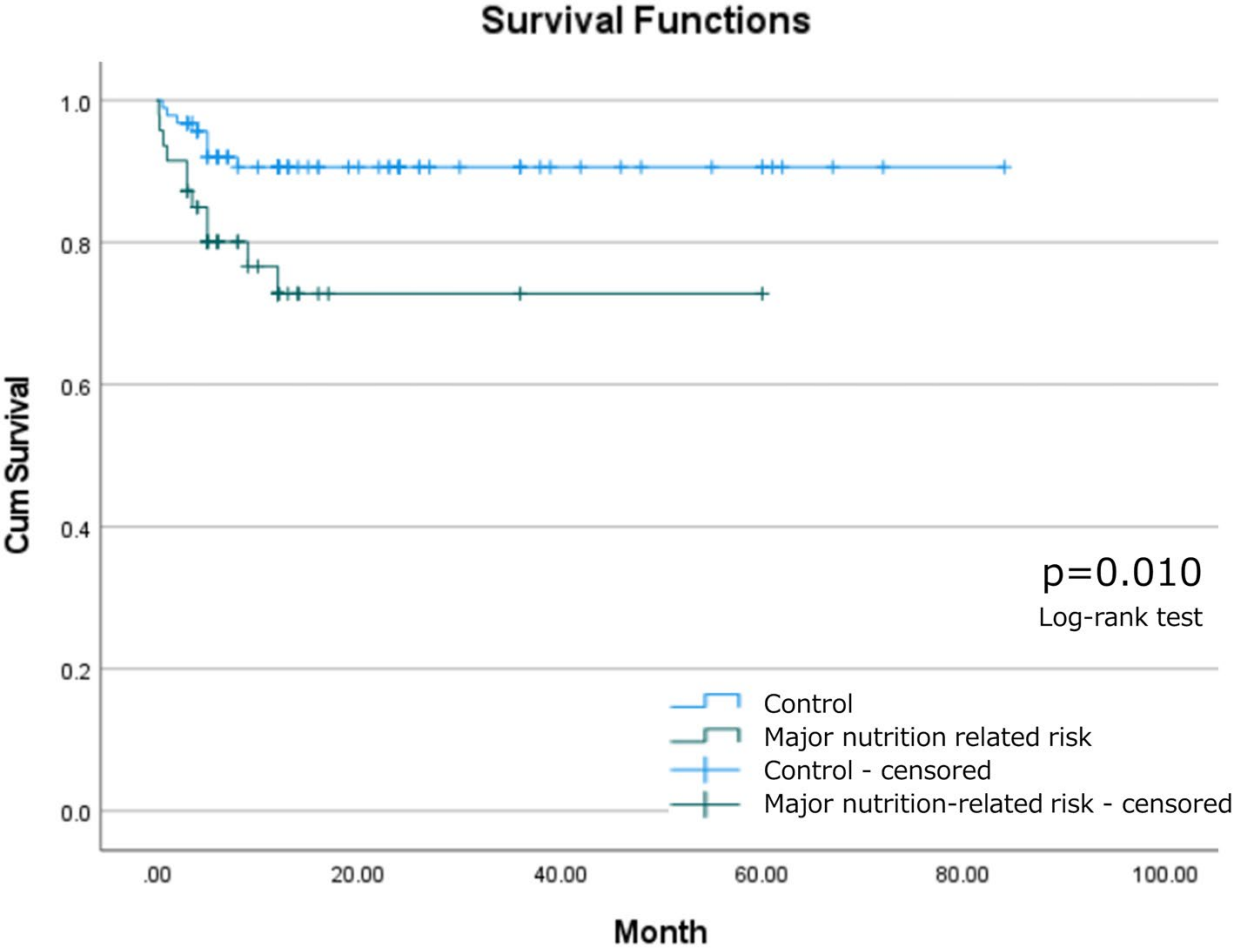
Survival analysis of patients with and those without major nutrition-related risk.

Among patients with major nutrition-related risks and matched controls, 11 (23.4%) and 8 (8.5%) patients died within 1-year post-trauma, respectively. The 1 year mortality ratio was significantly higher among patients with major nutrition-related risk than among matched controls (p = 0.019, Fig. 1). Per the Kaplan–Meier analysis, the median survival time was 44.9 [95% confidence interval (CI) 37.1–52.8] and 76.5 (95% CI 71.5–81.5) months for patients with major nutrition-related risk and matched controls, respectively. Patients with major nutrition-related risk survived for a significantly shorter period after experiencing cervical SCI than matched controls (Fig. 2, p = 0.015).

### Neurological improvement and ADL

In the subgroups of patients with AIS grades A–C on admission, the matched controls had a significantly higher proportion of patients who achieved a neurological improvement of at least one AIS grade than patients with major nutrition-related risk (57.1% vs. 34.5%, p = 0.039, Table 4). Specifically, 6, 3, and 1 patient among individuals with major nutrition-related risk and 22, 4, and 6 individuals among matched controls improved their neurological impairment from AIS grades from A to B, from B to C, and from C to E, respectively. Meanwhile, no significant differences were observed between patients with major nutrition-related risk and matched controls in the subgroup of patients with AIS grade D on admission (11.1% vs. 18.4%, p = 0.393). Regarding the ADL at 1 year post-injury, the ratios of the independent patient and wheelchair/bedridden individual were significantly lower and higher, respectively, in patients with major nutrition-related risk than in those of matched controls. (overall: p = 0.016, residual analysis: p < 0.05 respectively).

**Table 4:** Comparisons of neurological improvement and ADL at 1 year post-injury.

	Patients with major nutrition-related risk	Matched control	p value
AIS grade A, B, or C on admission			0.039^#^
Improved ≥ 1 grade	10 (34.5)	32 (57.1)	
Not improved or dead	19 (65.5)	24 (42.9)	
AIS grade D on admission			0.393^#^
Improved ≥ 1 grade	2 (11.1)	7 (18.4)	
Not improved or dead	16 (88.9)	31 (81.6)	
ADL at 1-year post-injury			0.016^#^
Independent	6 (15.0)	32 (39.0)	< 0.05^‡^
Walk with assistance	16 (40.0)	29 (35.4)	
Wheelchair/bedridden	18 (45.0)	21 (25.6)	< 0.05^‡^

*ADL* activities of daily living, *AIS* American spinal injury association impairment scale.

^#^Chi-square test (one-sided), ^‡^residual analysis.

## Discussion

In this study, approximately 35% of the older patients with cervical SCI had nutrition-related risk. Particularly, 6% of these patients had severe malnutrition, which could have major nutrition-related risks. Such patients showed substantially higher mortality rates within 1 year, shorter survival times, poorer neurological improvement, and lower levels of ADL at 1 year post-injury than matched controls.

We used GNRI in this study, rather than a single BMI or serum albumin (Alb) level, to assess the nutritional status^15,16^. Although GNRI has not previously been reported for SCI, the index was used in nutrition and other areas, such as urology, geriatrics, and cardiology^17–19^. For example, Kobayashi et al. showed that the GNRI is a substantial predictor for mortality among patients undergoing hemodialysis^17^, while Ruan et al. reported that the GNRI can serve as an independent prognostic factor for the overall survival of older patients with cancer cachexia^18^. Additionally, Kawakubo et al. revealed that malnutrition assessed using the GNRI predicts long-term adverse outcomes among hospitalized patients with heart failure with reduced ejection fraction^19^. Following these studies, we revealed that the GNRI would be useful in evaluating older patients with traumatic SCI. Our study results also revealed that 35% of the enrolled participants had nutrition-related risk, which was clearly higher than that in the general older population^11^. Therefore, these results could encourage physicians to assess the GNRI of older patients with SCI on admission.

Regarding the nutritional status of patients with SCI, some studies have elucidated the relationship between malnutrition and clinical complications in this cohort^12^. Hypoproteinemia and malnutrition might be indicators of mortality in patients with SCI^13^, and BMI is correlated with the recovery of ADL after experiencing SCI^14^. In addition to these studies, our study demonstrated that malnutrition in older patients with SCI was considerably associated with increased mortality within 1 year and a poorer improvement of neurological impairment. The mortality rate was not unexpected because the GNRI was designed to identify nutrition-related risk, including survival rate, as was validated in many other pathologies described above. However, our study results might add to the knowledge that the GNRI would be useful for predicting mortality in older patients with SCI.

Additionally, this report might be the first to demonstrate the relationship between nutrition-related risk and improvement in neurological impairment. There are three postulated reasons for this result. First, malnutrition might prevent neurological improvement at the cellular level. Previous studies have demonstrated that patients with SCI do not need only macronutrients that produce energy but also micronutrients, such as vitamins and minerals, to ensure proper cellular health, water and nutrient transport, and acid–base balance^25^. Second, evidence shows that the older population with severe malnutrition tends to have a lower motivation for every activity^26^. Consequently, the lower motivation to improve neurological symptoms in the older population with malnutrition would prevent vigorous rehabilitation, resulting in poor clinical outcomes. Third, patients with malnutrition tended to have sarcopenia in the chronic phase, which resulted in poor musculoskeletal functional improvement and accelerated musculoskeletal atrophy^27^. Such reasons or other factors might be entangled with each other, resulting in poor neurological improvement and lower levels of ADL at 1 year post-injury in older patients with malnutrition after SCI.

Gater et al. published guidelines for providing appropriate diet and nutrition after SCI^28^. The summarized points are as follows. (1) Resting energy expenditure was determined every 1–3 years to ensure an accurate assessment of energy balance. (2) Body fat was annually assessed with an obesity surrogate of BMI ≥ 22.3 kg/m^2^. (3) Review of negative energy balance, targeted diets, and exercises needed for fat loss; lipid management; and target glycated hemoglobin (HbA1c). (4) The fasting lipid profile was assessed annually to achieve target levels of triglycerides of ≤ 150 mg/dL and high-density lipoprotein cholesterol of ≥ 40 mg/dL. (5) Assess fasting blood glucose and HbA1c levels every 3 years to achieve a target HbA1c level of < 7%. As the number of older people with malnutrition increases, assessing the nutritional status on admission becomes critically important^11^. Furthermore, SPINE20, which is the advocacy group to bring global attention to the burden of disability caused by spinal disorders, released a recommendation in 2022 stating, “SPINE20 calls upon G20 countries to create a competent workforce and improve the health care infrastructure/facilities, including equipment to provide evidence-based inter-professional services to patients with spinal cord injury throughout their continuum of care”^29^. Our study results validated the significance of their guide and statements that a multidisciplinary approach with a long-term aspect is essential for older patients with malnutrition and SCI to decrease the mortality rate and improve neurological recovery.

This study had some limitations that should be addressed. First, all data were retrospectively collected. Although patients without missing major data were registered in our database, we did not have information about the exact number of cases excluded from the database. Additionally, the treatment strategy was determined by each physician and may not have been consistent among patients. Second, the study population included only Japanese and was heterogeneous. Third, we did not evaluate nutritional intervention, which limits us from reaching a definitive conclusion on this topic. Fourth, in this study, we did not consider several potentially important factors for outcomes, such as the length of rehabilitation and patients’ comorbidities, except for diabetes. Therefore, based on our results, a prospective international multicenter study should be conducted to test the evidence-based clinical effectiveness and cost-effectiveness of nutritional intervention for older patients with SCI and malnutrition.

In conclusion, approximately 35% of the older patients with cervical SCI in our study had nutrition-related risks. Particularly, 6% of these patients had severe malnutrition with major nutrition-related event risks. They also exhibited considerably shorter survival times, higher 1 year mortality rates, poorer neurological improvement, and lower levels of ADL at 1 year post-injury than matched controls. Therefore, assessing nutrition-related risk for older patients with SCI, as well as a multidisciplinary approach with long-term follow-up for such patients with nutrition-related risk, will be essential to decrease the mortality rate and improve neurological recovery and ADL.

## Data Availability

The data that support the findings of this study are available from the corresponding author upon reasonable request.
